# Effect of intravitreal aflibercept on recalcitrant diabetic macular edema

**DOI:** 10.1186/s40942-017-0064-0

**Published:** 2017-04-03

**Authors:** Kendra A. Klein, Tina S. Cleary, Elias Reichel

**Affiliations:** 1 New England Eye Center and Tufts Medical Center, Tufts University, 260 Tremont Street, Biewend Building, 9 - 11th Floor, Boston, MA 02111 USA; 2 Ophthalmic Consultants of Boston, 50 Staniford Street, #600, Boston, MA 02114 USA

**Keywords:** Aflibercept, Diabetic macular edema, Anti-VEGF, Chronic

## Abstract

**Background:**

Despite anti-VEGF therapy, some patients develop chronic diabetic macular edema. The objective of this study was to evaluate anatomic and visual outcomes of switching patients with chronic DME from intravitreal bevacizumab or ranibizumab to intravitreal aflibercept injection.

**Methods:**

In this retrospective observational case series, 11 eyes with recalcitrant diabetic macular edema (DME) were evaluated 6 months prior to and 6 months following initial intravitreal aflibercept injection (IAI). Recalcitrant DME was defined as having a thickened retina (≥350 μm) on spectral domain optical coherence tomography (SD-OCT) with persistent cystic changes (less than a 15% reduction in central retinal thickness) over 6 months prior to intravitreal aflibercept switch despite aggressive treatment for DME during this time.

**Results:**

One hundred and forty-seven patients in total were treated with IAI during this time, and of these, 31 patients were treated with IAI for DME. 18 eyes had less than 4 treatments within the 6 months prior to switch to IAI, 6 patients had a central retinal thickness (CRT) on SD-OCT of less than 350 μm at time of switch to IAI, and 2 patients had a greater than 15% decline in CRT on SD-OCT over the 6 months prior to switch to IAI. A total of 11 patients were included in the study. Over the 6 months prior to switch, the mean change in central retinal thickness was +18.6% and over the 6 months following switch to aflibercept the mean change in central retinal thickness was −27.1%. Switching to a regimen of at least 3 intravitreal aflibercept injections over 6 months resulted in some anatomic improvement and improvement or stabilization of Snellen visual acuity in all eligible patients.

**Conclusions:**

In patients with recalcitrant diabetic macular edema, switching to intravitreal aflibercept resulted in improved a 25% or more decrease in central retinal thickness in 81% (9/11) patients at 6-month follow-up. Sixty-three percent (7/11) had improvement in Snellen visual acuity after switching to intravitreal aflibercept injection, suggesting some reversibility of functional damage.

## Background

Diabetic macular edema (DME) is the leading cause of central vision loss in people with diabetes and is characterized by edema and thickening of the macula [1]. From the 1980’s until recently, macular laser photocoagulation, as evidenced by the Early Treatment Diabetic Retinopathy Study (ETDRS), remained the gold standard treatment for DME against which newer therapies were compared [2]. Recently, better understanding of the pathophysiology of DME highlighting the pivotal role of vascular endothelial growth factor (VEGF) and the introduction of anti-VEGF intravitreal agents has revolutionized DME treatment [3]. In 2013, 90% of retinal specialists in the USA reported using anti-VEGF agents for initial management of DME [4].

Intravitreal ranibizumab (Lucentis; Genentech, San Francisco, California) became the first VEGF inhibitor FDA-approved for the treatment of DME in 2012 following the RIDE and RISE trials, which showed significant superiority of both 0.3 and 0.5 mg ranibizumab groups over sham injection in improving visual acuity and decreasing central retinal thickness in patients with DME [5]. Likewise, off-label use of intravitreal bevacizumab (Avastin; Genentech) has been shown to be effective in the treatment of DME through studies such as PACORES [6], BOLT [7], and a phase II trial put forth by the DRCR.net [8].

In July 2014, the FDA approved intravitreal aflibercept (Eylea; Regeneron, Tarrytown, New York), a highly specific fusion protein with high VEGF-binding affinity, for the treatment of DME. This approval followed the VIVID and VISTA clinical trials, which showed superiority in anatomic and visual outcomes in patients treated with intravitreal aflibercept in comparison to laser treated controls. Patients in these studies required a 3-month washout period for patients previously treated with anti-VEGF agents [9].

In March 2015, Protocol T of the Diabetic Retinopathy Clinical Research Network (DRCR.net) published results of a study comparing the relative efficacy of intravitreal bevacizumab, ranibizumab, and aflibercept in treatment of DME. They found that all three anti-VEGF treatments improved vision in center-involving DME, with the relative effect depending on baseline vision. In cases of mild vision loss (20/40 or better), there was no significant difference between the three treatment groups. However, in cases with poorer baseline visual acuity (20/50 or worse), aflibercept was significantly more effective than both ranibizumab and bevacizumab in improving vision. Although Protocol T provides data on the comparative effectiveness across the 3 anti-VEGF agents, this study was not designed to inform on the relative efficacy when switching agents, and excluded eyes that received an anti-VEGF injection within the preceding 12 months [4].

Since FDA approval of aflibercept for the treatment of DME and results from Protocol T, many ophthalmologists are switching patients from ranibizumab or bevacizumab to aflibercept, especially those with minimal or incomplete response to the former treatments. In the current study, we evaluated the outcome of switching to aflibercept (IAI) in patients with DME considered to be “recalcitrant” to therapy. Patients were deemed recalcitrant if they had minimal decrease in central retinal thickness (CRT) on spectral domain optical coherence tomography (SD-OCT) despite aggressive treatment during the prior 6 months.

## Methods

A retrospective, observational case series was performed to study the outcome of switching to intravitreal aflibercept in eleven eyes with DME who were considered to be sub-optimal responders to standard of care and were considered to be “recalcitrant” to current therapy. The Institutional Review Board at Tufts Medical Center approved the study protocol for human subjects. The study was complaint with the Health Insurance Portability and Accountability Act of 1996 and adhered to the tenets of the Declaration of Helsinki.

Intravitreal injection logs for all patients treated with intravitreal aflibercept 2.0 mg (0.05 mL) at two clinical sites of the Vitreoretinal Service at Tufts Medical Center Department between March 2014 and July 2015 were reviewed. Electronic medical records were then reviewed to identify possible participants that were treated with intravitreal aflibercept injection for DME.

Inclusion criteria included ability to provide written informed consent, age 18 years or older, adequately clear media for SD-OCT imaging, and Snellen visual acuity of 20/40 to 20/300 in the study eye. Anatomic criteria for inclusion required CRT ≥ 350 μm on SD-OCT and recalcitrant DME was defined as persistent cystic change with ≤15% decrease in CRT over the 6 months prior to IAI switch despite having at least 4 total treatments for DME, with at least 3 of these treatments being intravitreal anti-VEGF injections (excluding IAI). Treatments for DME prior to IAI switch included intravitreal bevacizumab (IVB) and ranibizumab (IVR), intravitreal triamcinolone acetonide (IVTA), sub-Tenon’s triamcinolone acetonide (STTA), dexamethasone intravitreal implant (Ozurdex; Allergan, Irvine, California), and laser photocoagulation. If both eyes of a patient met entry criteria, then the worse-seeing eye was included in the study. If patients were treated with previous corticosteroids, baseline intraocular pressure had to be 21 mm Hg or less either with or without pressure reducing drops. Those patients who were previously treated with corticosteroids also had to have adequate clarity of media to allow adequate SD-OCT image quality. Patients were excluded if they had previously received intravitreal aflibercept in the study eye or a history of systemic anti-VEGF therapy. Inclusion and exclusion criteria are outlined in Table 1.

**Table 1:** Pre-defined inclusion and exclusion criteria

Inclusion criteria
Able to provide informed consent
Age 18 years or older
Clear ocular media
Baseline IOP of 21 mmHg or less with or without pressure-lowering drops in patients previously treated with corticosteroids
Snellen visual acuity between 20/40 and 20/300
CRT on SD-OCT ≥350 μm at the time of switch to IVE
Minimum of 4 treatments for DME, with at least 3 being anti-VEGF intravitreal injections (excluding IAI), administered within the 6 months prior to IAI switch
<15% decrease in CRT during the 6 months prior to IAI switch (worsening)
3 or more IAI within 6 months after switch
Exclusion criteria
Previous IAI in the study eye
History of systemic anti-VEGF use

Intravitreal injections were carried out using the same standard procedure in all patients. Dosing of intravitreal aflibercept was administered on a monthly as needed (PRN) regimen following the initial dose of aflibercept. The PRN treatment algorithm for DME entailed treatment with intravitreal aflibercept and monthly monitoring with SD-OCT. If at any point the central macula had no fluid at all, further aflibercept injections were withheld and the patient was monitored on a monthly basis. If new or increase in fluid occurred from the previous visit secondary to worsening of diabetic macular edema, re-treatment with intravitreal aflibercept was performed.

If the macula continued to have fluid after a minimum of 3 aflibercept injections and the macula achieved a 15% or more reduction in CRT compared to baseline on SD-OCT, then treatment could be withheld. If the macula continued to have fluid after a minimum of 3 aflibercept injections and the macula achieved <15% reduction in CRT compared to baseline of SD-OCT, aflibercept was continued or an alternative therapy was considered (corticosteroids or laser). If alternative therapy was utilized, the patient was not included in this analysis.

Patient charts were reviewed until 6 months after the first aflibercept injection. At each visit, best-corrected distance visual acuity was measured using a standardized Snellen chart, a comprehensive eye examination was performed including intraocular pressure measurement via applanation, and OCT imaging was obtained using Spectralis OCT (Heidelberg Engineering, Carlsbad, California, USA) or Cirrus HD-OCT (Carl Zeiss Meditech, Dublin, California, USA).

The occurrence of any severe postoperative complications, including infection, inflammation, retinal detachment, thromboembolic events, or death within the first 6 months after the first aflibercept injection was recorded.

## Results

The injection logs were reviewed for all patients receiving IAI between March 1, 2014 and July 1, 2015. One hundred and forty-seven patients in total were treated with IAI during this time, and of these, 31 patients were treated with IAI for DME. Twenty patients were excluded from the study for the following reasons: 18 eyes had less than 4 treatments within the 6 months prior to switch to IAI, 6 patients had a CRT on SD-OCT of less than 350 μm at time of switch to IAI, and 2 patients had a greater than 15% decline in CRT on SD-OCT over the 6 months prior to switch to IAI. A total of 11 patients with DME meeting inclusion criteria for the study were evaluated. The mean patient age was 65 years old (range 47–83 years). There were 7 men (64%) and 4 women (36%), and 5 (45%) right eyes and 6 (55%) left eyes included in the study. The self-reported mean hemoglobin A1c was 7.2 (range 6.1–10) and the mean duration of diabetes was 15 years (range 5–34 years) at the time of enrollment in the study. Baseline characteristics are outlined in Table 2.

**Table 2:** Demographics and clinical characteristics of patients with DME switched to IAI

**#**	Age (years)	Gender	Study eye	HBAlc (%), self-reported	Duration DM (years), self-reported
1	78	M	OD	7	20
2	64	M	OD	6.1	5
3	47	M	OS	8	15
4	47	M	OS	7.6	34
5	65	F	OS	6.4	5
6	62	M	OD	10	7
7	83	M	OS	6.5	20
8	64	F	OS	6.5	30
9	80	F	OD	7	15
10	54	F	OD	8	6
11	66	M	OS	6.6	13
	Mean = 65			Mean = 7.2	Mean = 15

### Treatment characteristics

The average duration of treatment duration for DME prior to inclusion in the study was 32 months (range 8–77 months). The mean total number of treatments given prior to inclusion in the study was 13 (range 5–30) and included intravitreal bevacizumab, intravitreal ranibizumab, intravitreal triamcinolone acetonide, sub-tenon triamcinolone acetonide, dexamethasone intravitreal implant, and focal laser. The anti-VEGF injections given prior to inclusion in the study included ranibizumab exclusively in 4 patients (36%), bevacizumab exclusively in 2 (18%) patients, and a combination of ranibizumab and bevacizumab in 5 (46%) patients. Other treatments included intravitreal triamcinolone acetonide (5 patients, 46%), sub-tenon triamcinolone acetonide (1 patient, 9%), focal laser (8 patients, 73%), and dexamethasone intravitreal implant (1 patient, 9%).

The average number of treatments for DME administered to the cohort during the 6 months before switch to IAI was 4.7 (range 4–6), with an average of 4.3 (range 3–6) being anti-VEGF injections. Eight patients (73%) were treated with ranibizumab exclusively, 2 patients (18%) were treated with bevacizumab exclusively, and 1 patient (9%) was treated with a combination of ranibizumab and bevacizumab. Other treatments included intravitreal triamcinolone acetonide (1 patient, 9%) and focal laser (4 patients, 36%). No patients received panretinal photocoagulation during this time.

During the 6 months after switch to IAI, an average of 4.7 (range 4–6) IAI were given. All patients had 6-month follow-up after switching to IAI and this visit was used for outcome analysis. Treatment characteristics are outlined in Table 3.

**Table 3:** Treatment characteristics prior to inclusion in the study, 6 months prior to IAI switch, and 6 months after IAI switch

*#*	Prior to inclusion in study			6 months prior to IAI switch		6 months after IAI switch
	Duration treatment (mo)	Total # treatment	Type of treatment	Total *#* treatment	Type of treatment	Total *#* IAI
l	60	26	6 IVB, 16 IVR, 2 IVTA, 1 STTA, 1 laser	5	5 IVR	4
2	18	10	4 IVB, 4 IVR, 1 IVTA, 1 laser	5	3 IVR, 1 IVTA, 1 laser	6
3	8	5	2 IVB, 3 IVR	4	1 IVB, 3 IVR	5
4	35	30	29 IVB, 1 laser	5	4 IVB, 1 laser	4
5	43	16	7 IVB, 8 IVR, 1 IVTA	5	5 IVR	6
6	9	5	4 IVR, 1 laser	5	4 IVR, 1 laser	4
7	19	16	16 IVR	5	5 IVR	5
B	36	18	6 IVB, 7 IVR, 2 IVTA, 1 dexamethasone intravitreal implant, 2 laser	6	6 IVR	4
9	10	6	4 IVR, 1 IVTA, 1 laser	4	3 IVR, 1 laser	5
10	77	7	5 IVR, 2 laser	4	4 IVR	4
11	33	6	5 IVB, 1 laser	4	4 IVB	5
	Mean = 32	Mean = 13	Mean # anti-VEGF = 11.5	Mean = 4.7	Mean # anti-VEGF = 4.3	Mean = 4.7

### Anatomic outcomes

The average CRT as measured on SD-OCT at 6 months prior to switch to IAI was 428 μm (range 299–690). The mean change in CRT during the 6 months prior to switch to IAI was an 18.6% increase. The average CRT at time of switch to IAI was 487 μm (range 363–685). At 6 months following switch to IAI, the average CRT was 326 μm (range 262–454). The mean change in CRT during the 6 months after switch to IAI was a 27.1% decrease. Anatomic and Snellen visual acuity outcomes before and after switch to IAI are outlined in Table 4.

**Table 4:** Anatomic and Snellen visual acuity outcomes 6 months prior to IAI switch, at time of IAI switch, and 6 months after IAI switch

**#**	6 months prior to IVE switch			Time of IVE switch		6 months after IVE switch		
	CRT(μ)	Change in CRT (%)	Snellen visual acuity	CRT (μ)	Snellen visual acuity	CRT (μ)	Charge in CRT (%)	Snellen visual acuity
1	299	+9.7	20/40	398	20/50	284	−23.1	20/40
2	490	+1.0	20/50	574	20/200	267	−44.3	20/70
3	496	−10.0	20/30	463	20/40	417	−5.7	20/25
4	313	+98.1	20/25	580	20/200	454	−34.4	20/200
5	460	+6.5	20/70	459	20/50	285	−34.5	20/50
6	384	+2.2	20/80	363	20/40	341	−5.7	20/40
7	383	+24.2	20/100	469	20/100	263	−40.0	20/50
8	690	+18.6	20/50	685	20/200	423	−13.2	20/200
9	353	+13.7	20/70	496	20/100	262	−41.9	20/70
10	347	+50.5	20/25	444	20/40	283	−36.4	20/25
11	498	−9.8	20/50	428	20/70	312	−18.6	20/40
	Mean = 428	Mean = +18.6%		Mean = 487		Mean = 326	Mean = −27.1%	

**Fig. 1:**
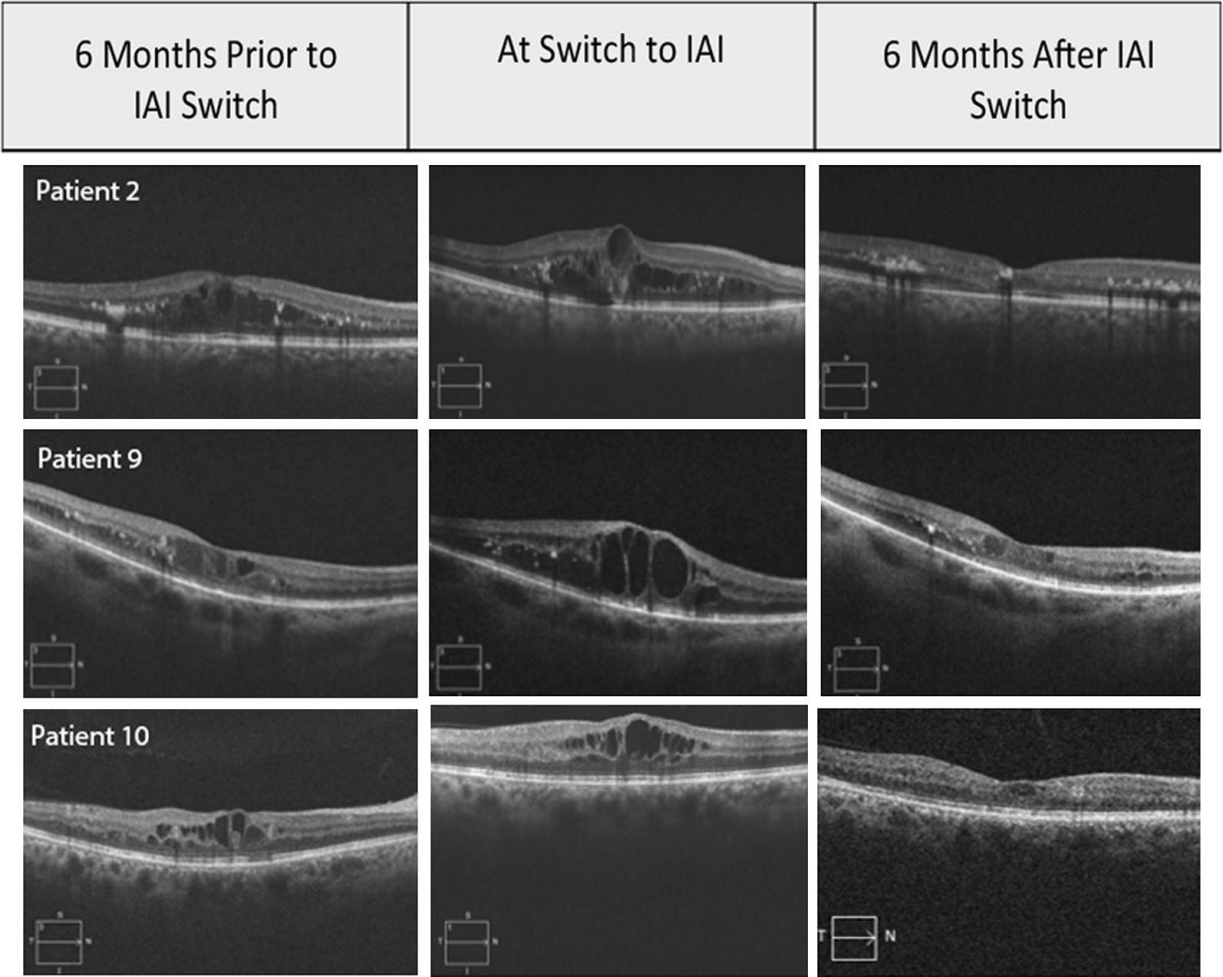
SD-OCT of patient 2, 9, and 10 at 6 months prior to IAI switch, at the time of IAI switch, and 6 months after IAI switch

At 6 months after switch to IAI, 9 of 11 patients (81%) had a 15% or more decrease in CRT, meeting the primary endpoint. At 6 months after switch to IAI, 9 of 11 patients (81%) had a 25% or more decrease in CRT and 1 of 11 patients (9%) had a 50% or more decrease in CRT. Eight of 11 patients (73%) had a CRT of less than 350 μm. Representative SD-OCT data before and after switch to intravitreal aflibercept is shown in Fig. 1.

### Visual outcomes

Six months prior to switch to IAI, the Snellen visual acuity ranged from 20/25 to 20/100. Over the 6 months prior to switch to IAI, the Snellen visual acuity declined in 8 patients (73%), improved in 2 patients (18%), and remained unchanged in 1 patient (9%). At time of switch to IAI, the Snellen visual acuity ranged from 20/40 to 20/200. Six months after switch to IAI, there was some improvement in Snellen visual acuity in 7 patients (63%) and remained stable in 4 patients (36%). No patients had decline in Snellen visual acuity after switch to IAI.

### Adverse events

After inclusion in the study, 52 anti-VEGF injections were given prior to switch, and 52 aflibercept injections were given after the switch. No ocular adverse events, including endophthalmitis, uveitis, retinal tears or detachment, or elevated intraocular pressure necessitating treatment were observed. There were no systemic adverse events such as thromboembolic phenomena (myocardial infarction, transient ischemic attack, or cerebrovascular accident) during the study.

## Discussion

Inflammatory changes in the retinal environment are involved in the pathogenesis of DME, including vascular leakage, leukostasis, ischemia, and release of pro-inflammatory mediators such as VEGF, interleukins 6 and 8, and TNF-alpha [10]. The retinal microenvironment in early DME may be characterized by acute inflammation and vascular dysfunction, whereas anatomic changes and neurotoxic effects from chronic edema may characterize long-standing DME. These unique characteristics found in chronic DME may alter response to various treatments [10, 11].

In clinical practice, some patients have chronic fluid, despite aggressive treatment with intravitreal injections of bevacizumab or ranibizumab. It is important to know if there is benefit to switching incomplete or non-responders to an alternative anti-VEGF agent, rather than continuing ranibizumab or bevacizumab injections, switching to corticosteroids, or combining anti-VEGF and corticosteroid treatment. The current study used stringent criteria identify patients with recalcitrant DME to evaluate if switching to IAI would be of benefit. In this small study, switching to IAI resulted in some anatomic improvement in all eligible patients, and 81% of patients met the pre-specified primary endpoint of 15% or greater decrease in CRT.

Our results are consistent with two previously reported cases series. Wood, Karth, Moshfeghi, and Leng evaluated short-term outcomes of switching to IAI in 14 eyes with persistent fluid from DME despite at least 3 monthly ranibizumab or bevacizumab. There was no minimum central retinal thickness requirement for inclusion and follow-up occurred after a single IAI. At 1 month after injection, 79% of patients had anatomic improvement on SD-OCT [12].

Rahimy et al. evaluated 50 eyes treated with at least 4 consecutive injections with ranibizumab or bevacizumab prior IAI switch, and two or more IAI after switch. Included patients had minimum central retinal thickness of 300 μm and follow-up occurred at month 2 or 3 after switch to IAI. At most recent follow-up, 56% had anatomic improvement on SD-OCT, while 24% had complete resolution of fluid [13].

The greater response to aflibercept in patients with chronic DME recalcitrant to treatment with bevacizumab and ranibizumab may be due to a number of factors, and may help explain the results seen in Protocol T. Aflibercept has been shown to have significantly higher binding affinity to VEGF-A compared to ranibizumab and bevacizumab. Also, aflibercept binds to VEGF-B and placental growth factor (PlGF), unlike ranibizumab and bevacizumab [9]. These characteristics may contribute to aflibercept’s superior effects on vascular permeability and retinal neovascularization, and may make it more suitable to treating patients with a high VEGF load, such as in chronic DME.

It is unclear both from inconsistencies in the published literature and clinically as to what parameters should be used to define recalcitrant DME (visual acuity response, anatomic response, number of treatments, et cetera). The current study used pre-defined and very stringent criteria for classifying patients as having recalcitrant DME, and included visual acuity, central retinal thickness, and treatment parameters. Recalcitrant patients were identified as those with substantially thickened retinas (>/350 μm CRT) with minimal improvement in CRT (<15% across 6 months prior to switch) despite aggressive therapy with at least four treatments for DME (3 of which were VEGFs other than aflibercept). These eyes were not improving anatomically prior to switch despite frequent anti-VEGF therapy. Switching to a pre-defined minimal use of aflibercept resulted in some anatomic improvement in all eligible patients. All patients were followed for at least 6 months, with the majority of patients gained some visual acuity after switching to aflibercept, suggesting some reversibility of functional damage.

The major limitations of this study include the retrospective design and the relatively small number of participants, precluding the ability to apply statistical analyses. Further studies are needed on a larger scale but this small, retrospective study suggests that switching to aflibercept can be beneficial for recalcitrant DME patients as defined in this study.

## Conclusions

In conclusion, in patients with recalcitrant DME, defined as having thickened retinas (≥350 μm) with minimal decrease in CRT (<15% over 6 months prior to switch) despite aggressive treatment (at least 4 treatments over 6 months prior to switch, 3 being anti-VEGFs), switching to intravitreal aflibercept may result in improved anatomic and visual outcomes at 6-month follow-up.

## Abbreviations

DME: diabetic macular edema
IVB: intravitreal bevacizumab
IVR: intravitreal ranibizumab
IAI: intravitreal aflibercept injection
IVTA: intravitreal triamcinolone acetonide
STTA: sub-Tenon’s triamcinolone acetonide
SD-OCT: spectral-domain optical coherence tomography
CRT: central retinal thickness
VEGF: vascular endothelial growth factor
PRN: pro re nata (as needed)
FDA: Food and Drug Administration
PLGF: placental growth factor
